# Multifractal analysis for all

**DOI:** 10.3389/fphys.2015.00027

**Published:** 2015-02-06

**Authors:** Peter Jurica

**Affiliations:** Cichocki Laboratory for Advanced Brain Signal Processing, RIKEN Brain Science InstituteSaitama, Japan

**Keywords:** multifractal, detrended fluctuation analysis, reproducibility, literate programming, MATLAB, Python

Multifractal formalism and analysis have been described many times (Lopes and Betrouni, 2009). There are several approaches to the implementation of multifractal analysis and there numerous ways to present these (Olsen, 1995; Wendt et al., 2009; Ihlen, 2012). Ihlen's article, in the June 4th 2012 issue of Frontiers of Physiology, entitled “Introduction to multifractal detrended fluctuation analysis in Matlab” provides a guide to the application of the method (hereafter MFDFA). The paper is one of many about the implementation of multifractal analysis. Here, I want to highlight the advantages of Ihlen's presentation and value of its approach for the sharing of scientific method in general. To give weight to my arguments, I provide a reproduction of the MFDFA implementation in Python.

Ihlen's presentation of MFDFA stands out from others in a number of respects. The article presents a recent and advanced concept, but it manages to do so at a level which anyone with basic MATLAB skills, independent of scientific background, should be able to follow. A similar paper would likely be found in the documentation of an MFDFA software library. However, Ihlen's paper appeared in a scientific journal. Thanks to this fact, and a proper peer-review, this “tutorial page” from a package's documentation offers a manual that is informative, easy to digest, yet is scientifically correct and elucidates a useful mathematical method of signal analysis to researchers without experience with complex mathematical notation.

Scientific method should not be a black box. The purpose of software in science is similar to the purpose of mathematics: it is a tool for the communication of ideas. Often, mathematics and software complement each other: while mathematics is more abstract and general, software is the choice for practical applications. Both methods of communication fulfill their roles in science only if their products are well understood. Understanding mathematical descriptions requires brainpower, years of education and practical application. In the case of software, many of the requirements can be “downloaded.” The download approach, however, opens up a new space for errors due to inappropriate application of the method. Such errors can often be avoided if the workings of a method are clear and well explained.

Is there a better way to teach a method than by demonstrating it, and its crucial properties, with real examples? The paper of Ihlen perhaps did not aim to follow the ideas of literate programming proposed by Knuth (1984), however it seems that it achieved some of the goals that Knuth and many others set for themselves about 30 years ago. The author explains MFDFA concepts from the ground up and backs every statement crucial to understanding and correct application of the method with a figure. Each of the figures in the publication can be obtained by copying, pasting and executing the code provided in the article. Another implementation of multifractal analysis by Wendt from IEEE Signal Processing Magazine (Wendt et al., 2009) is useful to draw the contrast between alternative approaches to method sharing. Wendt's article is independent of the toolbox's source code. As such, although correct, Wendt's implementation can be used without effort only as a black box. Ihlen's approach offers a second option: take only code snippets and compose your own implementation of MFDFA.

The tutorial approach worked well and helped me not only to adopt MFDFA in my analyses, but also to apply it across implementation language boundaries. To this end I have reproduced some of Ihlen's results in the Python programming language in the form of an interactive notebook (http://bsp.brain.riken.jp/~juricap/mdfa/mdfaintro.html), and in an extension module. The notebook's code can be interactively explored through Python's IPython package (Pérez and Granger, 2007), which is becoming a popular tool for keeping lab notes and sharing code with collaborators and reviewers (Shen, 2014). IPython notebooks use a web browser for their main user interface. Anyone interested in the package could start using it by downloading the notebooks, replacing the tutorial data files with their own, and exploring the method figure by figure. I believe that together with the text of the introduction to Ihlen's (2012) article, this package could make the MFDFA method accessible to all.

The approach of notebooks and literate programming has one important benefit for any single author who offers scientific software for reuse: the access to a proper code review. No serious business releases code without a proper review; its reputation and potential for customers depend on it. In research backed by software, it seems that only the correctness of results matter; how we get them is secondary. Very few reviews will ever venture beyond the proverbial double click and a visual confirmation of expected results. However, if the method is reused, currently it is most often reused through the software. It is thus the software, not just its verbal or mathematical description, which should be reviewed. This can be facilitated by making software code into an intrinsic part of an article, following Ihlen's approach or Knuth's suggestions. Interactive reviews are already possible, for example through interactive notebooks (Shen, 2014). Just as with published articles, public code sharing will drive authors toward a better style for source code and comments, and toward code that is not only correct, but also easy to parse and interpret.

## Conflict of interest statement

The author declares that the research was conducted in the absence of any commercial or financial relationships that could be construed as a potential conflict of interest.
